# Surgical planning of small intestine neuroendocrine tumors: the concept of mesenteric tumor deposits

**DOI:** 10.1530/EO-24-0056

**Published:** 2025-02-04

**Authors:** Romain L’Huillier, Gilles Poncet, Arnaud Pasquer, Thomas Walter, Catherine Lombard-Bohas, Valérie Hervieu, Bénédicte Cayot, Pierre-Jean Valette, Helen Cheung, Laurent Milot

**Affiliations:** ^1^ Department of Radiology, Hôpital Edouard Herriot, University Hospital of Lyon, Lyon, France; ^2^ Department of Digestive Surgery, Hôpital Edouard Herriot, University Hospital of Lyon, Lyon, France; ^3^ Department of Gastroenterology and Medical Oncology, Hôpital Edouard Herriot, University Hospital of Lyon, Lyon, France; ^4^ Department of Pathology, Hôpitaux Est, University Hospital of Lyon, Lyon, France; ^5^ Department of Medical Imaging, Sunnybrook Health Sciences Centre, Toronto, Canada

**Keywords:** neuroendocrine, small bowel, small intestine, computed tomography, mesenteric tumor deposits, surgical planning, lymph nodes, mesenteric mass, short small bowel syndrome

## Abstract

The mesenteric extension of small neuroendocrine tumors is the surgical limiting factor because of the risk of postoperative short bowel syndrome due to superior mesenteric artery involvement. Recent pathological studies have shown that this vascular involvement is due to mesenteric tumor deposits, differentiated from lymph node metastases. The aim of this study was to evaluate the performances of computed tomography (CT) for the surgical planning of small intestine neuroendocrine tumors. This was a retrospective observational study, and all patients undergoing surgery for small intestine neuroendocrine tumor between January 2014 and March 2019 were included. Preoperative CTs were reviewed, blinded from surgical and pathological data, by two radiologists. Diagnostic accuracy and interobserver reliability analysis were performed. We included 45 patients (mean age: 61 years (28–84 years); 23 men). The CT sensitivity to identify the mesenteric mass was 97% (37/38) with a *ĸ* of 0.73. The positive predictive value of CT to anticipate a right colic resection was 86% (18/21). The negative predictive value of CT was high (97% (34/35) to 100% (35/35)) for duodenal resection (*ĸ* = 0.78). Regarding retropancreatic lymph node invasion, the CT sensitivity was poor (24%, 4/17), with a high *ĸ* (0.88). The level of involvement by the mesenteric mass was correlated with the length and the percentage of the remaining small bowel. CT is essential for the surgical planning of small intestine neuroendocrine tumors, being accurate in defining the mesenteric tumor deposits, allowing one to anticipate, with a good reproducibility, the length and percentage of the remaining small bowel and the necessity for a right colectomy.

## Introduction

Small intestine neuroendocrine tumors (Si-NETs) are rare tumors with an estimated annual incidence of 1.2/100,000 in the United States (Scott & Howe 2018). They represent the first cause of malignant tumors of the small intestine (Dasari *et al.* 2017). Their prognosis is better than that of other tumor types with a survival rate of 95% at 5 years and 88.5% at 10 years (Wu *et al.* 2017). Si-NETs are mainly located in the terminal ileum (Keck *et al.* 2018) and are multiple in 30% of cases (Partelli *et al.* 2017).

Surgery is the only potentially curative treatment and should always be contemplated, even if liver or peritoneal metastases are present, as there are risks of intestinal ischemia and occlusion related to locoregional mesenteric tumoral extension (Partelli *et al.* 2017). Mesenteric tumor extension along the superior mesenteric vessels, especially the superior mesenteric artery (SMA), is the limiting factor for surgical resectability because of the risk of postoperative short small bowel syndrome (Pasquer *et al.* 2021). Until very recently, the mesenteric tumor extension of Si-NETs has exclusively been considered as lymphatic and the local operability assessment of Si-NETs has largely been based on the location of lymph nodes (LNs) along the superior mesenteric axis on preoperative imaging (Lardière-Deguelte *et al.* 2016). Recent anatomopathological studies have distinguished LN metastasis from mesenteric tumor deposits (MTDs) by the presence of entrapped nerves and arteries in MTDs. Irregular contours are frequent but not systematic (Gonzalez *et al.* 2018).

The surgical challenges related to the involvement of the SMA and its branches would therefore be due to the MTDs and not to the mesenteric LN metastasis. The presence of MTDs, noted in nearly 65% of cases, is also a predictive factor for the development of liver metastases and is associated with a decrease in survival (Fata *et al.* 2017), so much so that it has been included in the latest version of the American Joint Committee on Cancer (AJCC) Cancer Staging Manual (Amin *et al.* 2017). A MTD > 2 cm is called a mesenteric mass.

There is currently a trend to standardize the description of the mesenteric extension of neuroendocrine tumors of the small intestine on preoperative imaging (Dromain *et al.* 2022); however, there are no criteria for distinguishing MTDs from mesenteric LN metastases (Blažević *et al.* 2018). Moreover, even if duodenal invasion by MTDs is described (Moris *et al.* 2018) with the potential implication of a duodenal surgical resection, there is no radiological criterion defining duodenal involvement.

In addition, there are no criteria described to predict the need for an associated right colic resection, except the location of a Si-NET close to the ileocecal valve (Selberherr *et al.* 2017).

Abdominopelvic computed tomography (CT) is recognized as the reference imaging modality for the locoregional extension of Si-NETs (Lamarca *et al.* 2024).

In light of this new pathological classification of Si-NETs and the precise definition of the mesenteric mass, the aim of our study was to evaluate the performances of preoperative CT in the surgical planning of Si-NETs in order to predict tumor multiplicity, the presence of a mesenteric mass, the need for right colic or duodenal resection, retropancreatic LN involvement and the length and percentage of the remaining small bowel.

## Materials and methods

### Study design and patients

This was a single-center, retrospective, observational study of all patients who underwent surgery for Si-NETs at the ENETS Centers of Excellence (CoEs) of Lyon between January 1, 2014, and March 31, 2019. Patients with appendicular or duodenal neuroendocrine tumors were ineligible.

**Figure 1:**
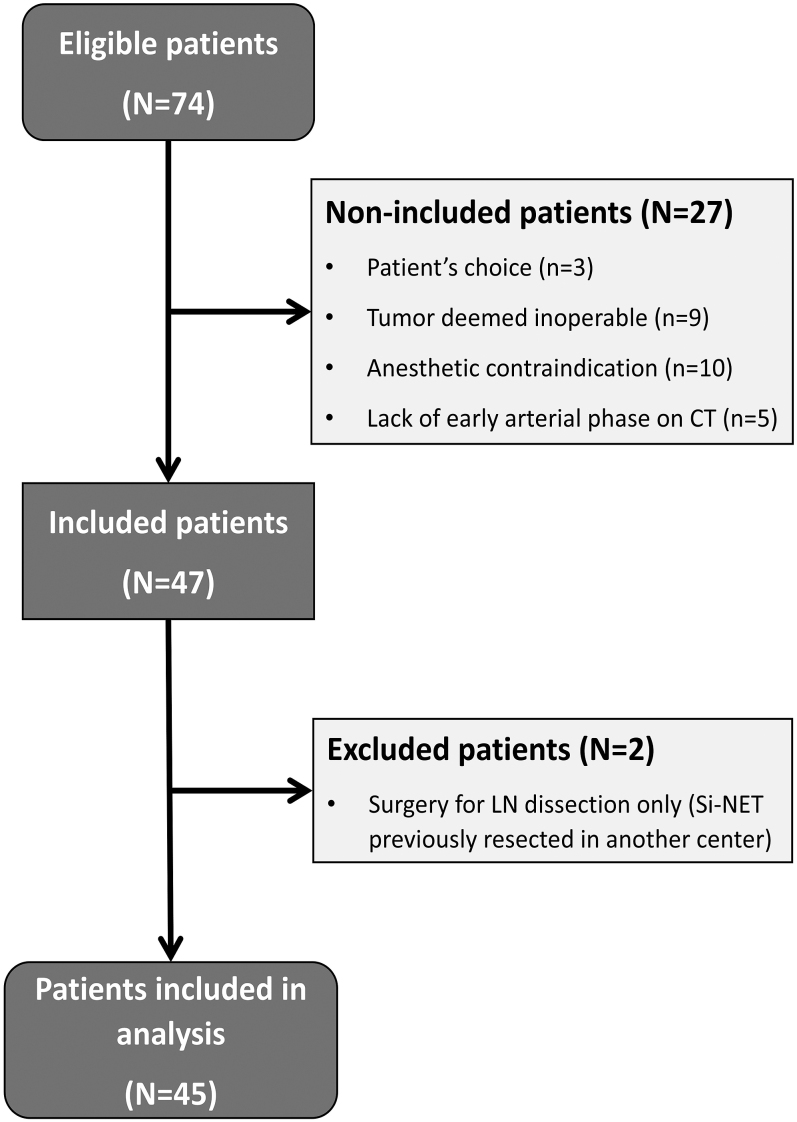
Flow chart of patient inclusion. CT, computed tomography; LNs, lymph nodes; Si-NET, small intestine neuroendocrine tumor.

We evaluated 74 patients: 27 patients were not included (ten because of anesthetic contraindication, nine because of inoperable tumor, five because of incomplete imaging and three because of patient’s choice); two patients were excluded because they were referred only for LN resection. Forty-five patients were included in the study (Fig. 1).

An information leaflet was sent to each patient, and their consent was collected. The study was approved by the institutional review board and has been inscribed on Clinical Trials Registry (NCT03958188).

Biological, surgical and pathological data were prospectively collected in the hospital database.

We collected the length of the resected small bowel and that of the remaining small bowel and the need for a right colectomy, a duodenal resection or resection of metastases.

All patients received retropancreatic LN dissection to prevent future unresectable disease by invasion of the SMA root (Pasquer *et al.* 2016).

Histological examinations were performed in the ENETS CoEs and concerned the primary tumor(s) (number, size, localization and KI67), mesenteric and retropancreatic LNs, MTDs, including the presence of a mesenteric mass (MTD > 20 mm), and metastases.

The latest version of the American Joint Committee on Cancer (AJCC) Cancer Staging Manual (Amin *et al.* 2017) was used for tumor staging.

### Test methods

The CTs were reviewed independently and blinded to surgical and pathological findings and also blinded to other radiological exams (e.g., somatostatin analog uptake studies), by two radiologists, one expert (LM – 20 years of experience) and a resident in training (BC). Each abdominopelvic CT included at least an early arterial and a portal phase. Native acquisitions (slices <1 mm) were available. All CT were reviewed on the same software (IntelliSpace Portal 11®). Multiplanar reconstructions, volume rendering technique and maximum intensity projection reconstructions, which are known to be very useful for tumor detection, mesenteric and vascular evaluation (Dohan *et al.* 2015), were available.

#### Si-NETs

**Figure 2:**
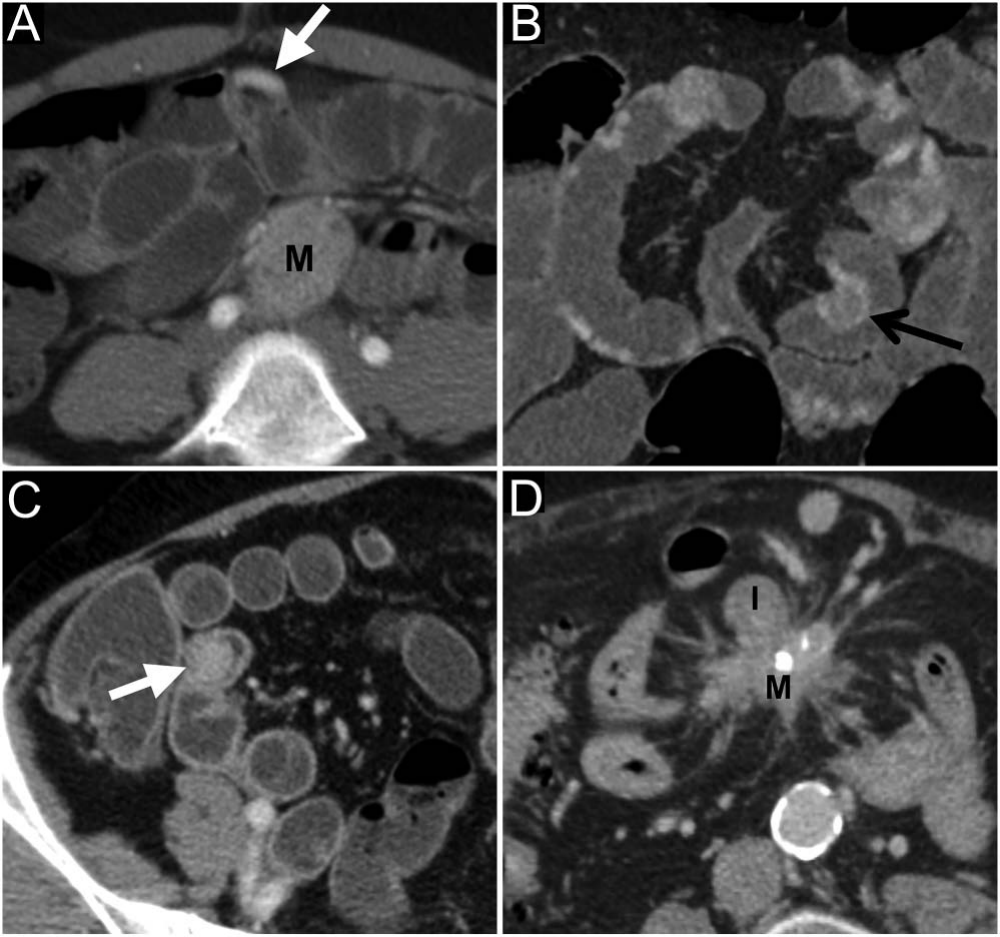
Different aspects of Si-NETs. M, mesenteric mass and I, ileum. (A) The patient is a 28-year-old woman. Arterial phase – axial. Ileal neuroendocrine tumor (white arrow) as a focal thickening of the ileum, hypervascular on arterial phase, with adjacent mesenteric mass (M). (B) The patient is a 58-year-old man. Arterial phase – coronal reconstruction – multiple small bowel neuroendocrine tumors, sometimes presenting a retractile character, creating a hairpin appearance (black arrow). (C) The patient is a 61-year-old woman. Portal phase – axial – enteroclysis. Endoluminal Si-NET (white arrow) as an enhanced nodular tissue lesion within the terminal ileum. (D) The patient is a 76-year-old man. Portal phase – axial. The retractile (and partially calcified) mesenteric mass (M) is in contact with the ileum (I), and the primary tumor is not visualized because of its fusion with the mesenteric mass.

The presence of Si-NET(s) was defined according to classic criteria (Bonekamp *et al.* 2015): arterially enhancing area(s) of focal nodular thickening with persistent enhancement during portal phase. The presence of a Si-NET on the left side of SMA trunk axis on coronal exploration was also reported because it would be an argument for Si-NET multiplicity (Kalifi *et al.* 2020). Different aspects of the Si-NETs are presented in Fig. 2.

#### Mesenteric mass (MTD>2 cm)

**Figure 3:**
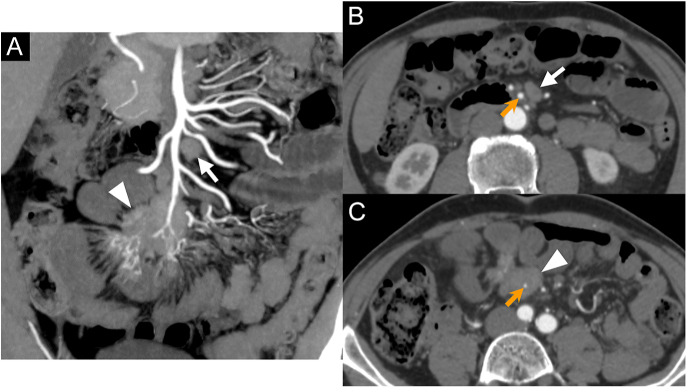
How to distinguish mesenteric lymph nodes metastases and mesenteric tumor deposits. Images A, B and C are from the same CT (arterial phase) in a 69-year-old man. (A) Coronal MIP 10 mm reconstruction at the arterial phase showing mesenteric mass (white arrowhead) involving the distal part of the SMA trunk and mesenteric lymphadenopathy (white arrow) located high up in the mesentery, close to the first jejunal branches. (B) Axial MPR 1 mm reconstruction at the level of the mesenteric lymphadenopathy (white arrow), which is close to the trunk of the SMA (orange arrow), but there is no contact (persistent fat plane between them). (C) Axial MPR 1 mm reconstruction at the level of the mesenteric mass (white arrowhead), which encases (contact with >180°) the trunk of the SMA (orange arrow).

Unlike metastatic LNs, MTDs involve the trunk or branches of the SMA (Fig. 3). Arterial involvement was defined by a contact >180°, in a perpendicular plane to the axis of the artery, based on the classic criteria for vascular involvement from pancreatic adenocarcinoma (Zins *et al.* 2018).

The location of the mesenteric mass within the mesentery, the total number of small bowel arteries and the number of involved and noninvolved arteries were systematically reported. These data were classified based on the level of arterial invasion along superior mesenteric axis by the mesenteric mass using Lardière-Deguelte *et al.*’s (2016) classification but focusing on the extent of the mesenteric mass rather than lymphadenopathy.

#### Right colic resection

**Figure 4:**
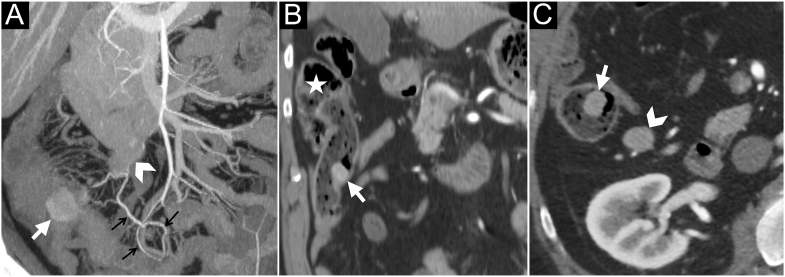
CT signs evaluated for the need of a right colectomy. (A) The patient is a 55-year-old woman. Arterial phase – coronal oblique MIP reconstruction. The mesenteric mass (arrowhead) involves the ileocolic artery, which is very irregular, and there is an arterial supply network (small black arrows) from the ileal branches. The Si-NET (white arrow) is located near the ileocecal valve. (B) The patient is a 66-year-old man. Arterial phase – coronal reconstruction. The Si-NET (white arrow) is located near (<10 cm) the ileocecal junction and the cecum (white star). (C) The patient is a 66-year-old man. Arterial phase – axial. Presence of a LN metastasis in the right mesocolon (white arrowhead), adjacent to a distal Si-NET (white arrow).

Three signs were tested (Fig. 4): the presence of a Si-NET close to the ileocecal valve (<10 cm), involvement of the ileocolic artery by the mesenteric mass and the presence of right mesocolon LN metastases (based on the existence of at least one of the following three criteria, described in previous studies: small axis >10 mm (Charnsangavej *et al.* 1993), round shape (Ganeshalingam & Koh 2009) and early enhancement (Lardière-Deguelte *et al.* 2016)).

The presence of one sign was sufficient.

#### Duodenal resection

**Figure 5:**
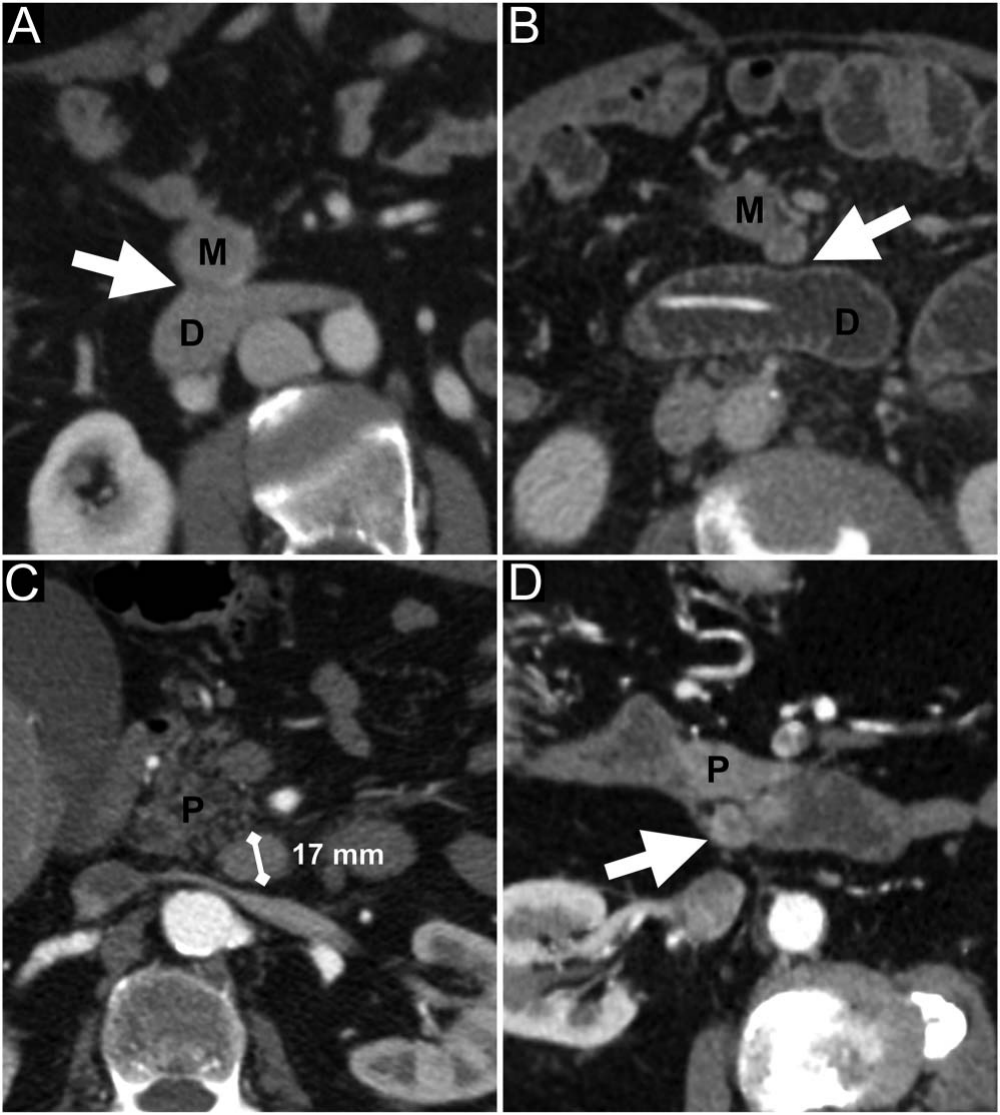
Duodenal and retropancreatic analysis on CT. M, mesenteric mass; D, duodenum; and P, pancreas. (A) The patient is a 55-year-old woman. Portal phase – axial. The white arrow shows the loss of the fat plane between the mesenteric mass (M) and the duodenum (D). (B) The patient is a 64-year-old man. Portal phase – axial – enteroclysis. The white arrow shows the persisting fatty plane between the mesenteric mass (M) and the duodenum (D). (C) The patient is a 76-year-old man. Arterial phase – axial. Retropancreatic lymphadenopathy retained as pathological because of the small axis >10 mm and the enhancement on arterial phase. (D) The patient is a 57-year-old man. Arterial phase – axial. Retropancreatic lymphadenopathy (white arrow) retained as pathological because of the enhancement on arterial phase and the round shape.

When a mesenteric mass was located in the proximal mesentery, its contact with the duodenum (loss of the fat plane) was systematically noted (Fig. 5).

#### Retropancreatic LN involvement

Retropancreatic LN corresponds to the distal lymphatic drainage at the origin of the superior mesenteric vessels (Pasquer *et al.* 2016).

We evaluated the performances of CT in the detection of retropancreatic LN invasion, based on the same criteria used for right mesocolon LN metastases: small axis >10 mm (Charnsangavej *et al.* 1993), round shape (Ganeshalingam & Koh 2009) and early enhancement (Lardière-Deguelte *et al.* 2016) (Fig. 5).

#### Length and percentage of the remaining small bowel

The associations between the involvement of the SMA trunk, the number and the percentage of noninvaded small bowel arteries and the length and percentage of the remaining small bowel were evaluated.

Moreover, both raters were asked to subjectively estimate the percentage of the remaining small bowel at the end of surgery.

### Statistical analysis

Sensibility, specificity, positive predictive value (PPV) and negative predictive value (NPV) were evaluated with a 95% CI and calculated using 2 × 2 contingency tables. We used the kappa coefficient to evaluate the interobserver reliability.

We used the Mann–Whitney test to evaluate the correlation between the involvement of SMA trunk and the length of the remaining small bowel and Spearman’s correlation for the association between the number and the percentage of small bowel arteries noninvolved by the mesenteric mass and the length and percentage of the remaining small bowel. We used an intraclass correlation coefficient (ICC) to compare the subjective estimation of the raters with surgery concerning the percentage of the remaining small bowel. An inter-rater reliability analysis was performed, using kappa coefficient concerning the involvement of SMA and using an ICC concerning the estimation of the remaining length of small bowel after surgery and the number and percentage of noninvolved small bowel arteries. Statistical analyses were performed using SPSS version 24. A two-sided *P* < 0.05 was considered statistically significant.

## Results

### Patient characteristics

The population was composed of 23 men and 22 women, and the average age was 61 (SD = 11 years) (Table 1). Fifteen patients (33%) presented with flushing, 13 patients (29%) presented with diarrhea, and 26 patients (58%) suffered from abdominal pain. Eight patients (18%) had carcinoid heart disease. The average time between the preoperative CT and the surgery was 36 days (SD = 9 days). The average length of resected small bowel was 184 cm, and the average length of the remaining small bowel was 431 cm. One patient had a small bowel length after surgery (<200 cm). Moreover, 25 patients (56%) underwent right colic resection and four patients (9%) received additional duodenal resection. No patient died during surgery. No patient had a complication that required revision surgery.

**Table 1:** Patient characteristics.

Parameters	All patients (*n* = 45)
Age (years)	61 (28–84)
Gender – male	23 (51)
Symptoms	
Abdominal pain	26 (58)
Small bowel obstruction	3 (7)
Anemia/bleeding	5 (11)
Flush	15 (33)
Diarrhea	13 (29)
Weight loss	7 (16)
Carcinoid heart disease	8 (18)
Urinary 5-HIAA (μmol/24 h)	155.5 (5–969)
CT	
Early arterial phase on abdomen and pelvis	45 (100)
Portal phase on abdomen and pelvis	45 (100)
Enteroclysis	6 (13)
Surgery	
Length of the resected small bowel (cm)	184 (25–520)
Length of the remaining small bowel (cm)	431 (185–700)
Length of the remaining small bowel <200 cm	1 (2)
Percentage of the remaining small bowel	71 (34–94)
Right colectomy	25 (56)
Duodenal resection	4 (9)
Other carcinological resections	30 (67)
Pathological findings	
Multiplicity of Si-NETs	23 (51)
Si-NET size (largest one when multiple) (mm)	18 (4–50)
Mesenteric mass	38 (84)
Number of mesenteric LNs removed	32 (8–86)
Number of mesenteric LNs involved	7 (0–29)
Ratio of mesenteric LNs involved/mesenteric LNs removed (%)	0.26 (0–0.73)
Retropancreatic LN involvement	17 (38)
Right mesocolic LN involvement	18 (40)
Peritoneal metastases	19 (42)
Liver metastases	31 (69)
Ovarian metastases	5 (23)
KI67 (%)	1.61 (0.4–20)
pT (UICC)	
pT1	2 (4)
pT2	4 (9)
pT3	34 (76)
pT4	5 (11)

CT, computed tomography; Si-NET, small intestine neuroendocrine tumor, LN, lymph node, UICC, Union for International Cancer Control. Quantitative variables are presented with the mean (except for Ki, for which it is the median) and extreme values in parentheses, while qualitative variables are presented with the number of patients and the corresponding percentage in parentheses. Information about the race of the patients is not available because its collection is not allowed in France.

One patient presented with a thrombosis of the superior mesenteric vein five days after surgery, which resolved after three months of curative anticoagulation.

Among patients included, 23 (51%) had multiple Si-NETs and 84% of patients were diagnosed with a mesenteric mass. On average, 32 mesenteric LN were resected, with an involved/removed LN ratio of 0.26. Finally, 17 patients (38%) had retropancreatic LN involvement and 31 patients (69%) had liver metastases.

### Main CT diagnostic performances

#### Multiplicity of Si-NETs

The sensitivity for the detection of ≥2 Si-NETs was 61% for the senior rater (95% CI: 39–80%) and 70% for the resident rater (95% CI: 47–86%), and the PPV was 78% for the senior rater (95% CI: 52–93%) and 67% (95% CI: 45–84%) for the resident rater (Table 2). Moreover, the agreement between the two raters was substantial with a kappa coefficient (*ĸ*) of 0.65 (95% CI: 0.43–0.87).

**Table 2:** Main CT diagnostic performances.

Variables	Senior rater	Resident rater	ĸ
Multiplicity of Si-NETs			
≥2 Si-NETs detected			0.65 [0.43–0.87]
Sensitivity	14/23 (61%) [39–80%]	16/23 (70%) [47–86%]	
Specificity	18/22 (82%) [59–94%]	14/22 (64%) [41–82%]	
PPV	14/18 (78%) [52–93%]	16/24 (67%) [45–84%]	
NPV	18/27 (67%) [46–83%]	14/21 (67%) [43–85%]	
Si-NETs on the left side of SMA trunk			0.21 [0–0.55]
Sensitivity	9/23 (39%) [20–61%]	10/23 (44%) [24–65%]	
Specificity	18/22 (82%) [59–94%]	21/22 (95%) [75–100%]
PPV	9/13 (69%) [39–90%]	10/11 (91%) [57–100%]	
NPV	18/32 (59%) [38–73%]	21/34 (62%) [44–77%]	
Presence of a mesenteric mass			0.73 [0.44–1]
Sensitivity	37/38 (97%) [85–100%]	37/38 (97%) [85–100%]	
Specificity	5/7 (74%) [30–95%]	6/7 (86%) [42–99%]	
PPV	37/39 (95%) [81–99%]	37/38 (97%) [84–100%]	
NPV	5/6 (83%) [36–99%]	6/7 (86%) [42–99%]	
Right colic resection			1 [1–1]
Sensitivity	18/25 (72%) [50–87%]	18/25 (72%) [50–87%]	
Specificity	17/20 (85%) [61–96%]	17/20 (85%) [61–96%]	
PPV	18/21 (86%) [63–96%]	18/21 (86%) [63–96%]	
NPV	17/24 (71%) [49–87%]	17/24 (71%) [49–87%]	
Duodenal resection			0.78 [0.55–1]
Sensitivity	4/4 (100%) [40–100%]	3/4 (75%) [22–99%]	
Specificity	36/41 (88%) [73–95%]	34/41 (83%) [67–92%]	
PPV	4/9 (44%) [15–77%]	4/10 (30%) [8–65%]	
NPV	36/36 (100%) [88–100%]	34/35 (97%) [83–100%]	
Retropancreatic LN involvement			0.88 [0.64–1]
Sensitivity	4/17 (24%) [8–50%]	4/17 (24%) [8–50%]	
Specificity	28/28 (100%) [85–100%]	27/28 (96%) [80–100%]	
PPV	4/4 (100%) [40–100%]	4/5 (80%) [30–99%]	
NPV	28/41 (68%) [52–81%]	27/40 (68%) [51–81%]	

Si-NET(s), small intestine neuroendocrine tumor(s); SMA, superior mesenteric artery; LNs, lymph nodes; PPV, positive predictive value; NPV, negative predictive value; *ĸ*, Cohen’s kappa coefficient. Results of each rater are presented in ratios with the corresponding percentages and 95% confidence intervals after. The Cohen’s kappa coefficient is given with its 95% confidence interval.

The presence of a Si-NET on the left side of SMA trunk had very low sensitivity, with 39% for the senior rater (95% CI: 20–61%) and 44% for the resident rater (95% CI: 24–65%). The inter-rater reliability was slight to fair with *ĸ* = 0.21 (95% CI: 0–0.55).

#### Presence of a mesenteric mass

Based on our criteria to distinguish mesenteric LN metastases from MTDs (Fig. 3), the sensitivity of CT to detect a mesenteric mass was 97% (95% CI: 85–100%) for both raters, with a PPV of 95% (95% CI: 81–99%) for the senior rater and 97% (95% CI: 84–100%) for the resident rater. Moreover, the *ĸ* was 0.73 (95% CI: 0.44–1).

#### Right colic resection

The three CT signs tested to assess the need for a right colic resection are shown in Fig. 4. The results were equal for both raters, with a kappa coefficient of 1. The sensitivity of CT to predict right colic resection was 72% (95% CI: 50–87%), the specificity was 85% (95% CI: 61–96%), the PPV was 86% (95% CI: 63–96%), and the NPV was 71% (95% CI: 49–87%).

#### Duodenal resection

The CT finding tested was the loss of the fat plane between the mesenteric mass and the duodenum (Fig. 5). Only four patients required duodenal resection. The PPV of the CT was low, with 44% (95% CI: 15–77%) for the senior rater and 30% (95% CI: 8–65%) for the resident rater, but the NPV was very high, with 100% (95% CI: 88–100%) for the senior rater and 97% (95% CI: 83–100%) for the resident rater. The inter-rater reliability was substantial with *ĸ* = 0.78 (95% CI: 0.55–1).

#### Retropancreatic LN involvement

Using our criteria (Fig. 5), the sensitivity of CT was 24% (95% CI: 8–50%) for both raters and specificity was 100% (95% CI: 85–100%) for the senior rater and 96% (95% CI: 80–100%) for the resident rater. The PPV was 100% (95% CI: 40–100%) for the senior rater and 80% (95% CI: 30–99%) for the resident rater, and the NPV was 68% (95% CI: 52–81%) for the senior rater and 68% (95% CI: 51–81%) for the resident rater. The inter-rater reliability was almost perfect, with *ĸ* = 0.88 (95% CI: 0.64–1). Examples of retropancreatic LN involvement are shown in Fig. 5.

### CT performances concerning the length and percentage of the remaining small bowel after surgery

#### Length of the remaining small bowel

For both raters, three variables (the involvement of the SMA trunk, the number and the percentage of noninvaded small bowel arteries) were statistically correlated with the length of the remaining small bowel after surgery (*P* < 0.05) (Table 3). Regarding the number of noninvolved SMA small bowel branches, Spearman’s correlation coefficients were positive for both raters (*ρ* = 0.44 for the senior rater and *ρ* = 0.56 for the resident rater). Similarly, Spearman’s correlation coefficients concerning percentages of noninvolved SMA small bowel branches were also positive (*ρ* = 0.47 for the senior rater and *ρ* = 0.51 for the resident rater).

**Table 3:** CT performances in assessing the length and percentage of the remaining small bowel.

CT assessment of the length of the remaining small bowel			
Variables	Senior rater	Resident rater	Inter-rater reliability
Involvement of SMA trunk by a mesenteric mass	Yes: 20/45	Yes: 24/45	Cohen’s kappa coefficient
	No: 25/45	No: 21/45	*ĸ* = 0.87 [0.72–1]
	*P* = 0.003	*P* = 0.001	
Number of SMA SB branches noninvolved	Correlation coefficient (*ρ*) = 0.44	Correlation coefficient (*ρ*) = 0.56	ICC = 0.80 [0.65–0.89]
	*P* = 0.005	*P* < 0.001	
Percentage of SMA SB branches noninvolved	Correlation coefficient (*ρ*) = 0.47	Correlation coefficient (*ρ*) = 0.51	ICC = 0.82 [0.68–0.90]
	*P* = 0.003	*P* = 0.001	

CT assessment of the percentage of the remaining small bowel			
Variables	Senior rater	Resident rater	Inter-rater reliability
Number of SMA SB branches noninvolved	Correlation coefficient (*ρ*) = 0.51	Correlation coefficient (*ρ*) = 0.68	ICC = 0.80 [0.64–0.89]
	*P* = 0.001	*P* < 0.001	
Percentage of SMA SB branches noninvolved	Correlation coefficient (*ρ*) = 0.74	Correlation coefficient (*ρ*) = 0.63	ICC = 0.82 [0.69–0.90]
	*P* < 0.001	*P* < 0.001	
Rater’s personal estimation of small bowel % remaining	ICC = 0.64 [0.42–0.78]	ICC = 0.50 [0.25–0.70]	ICC = 0.58 [0.34–0.74]

CT, computed tomography; SMA, superior mesenteric artery; SB, small bowel; ICC, intraclass correlation coefficient. For categorical variables, the ratios are presented for each rater with the *P*-value result of the statistical test. For continuous variables, the correlation coefficient is presented with the *P*-value result of the statistical test. For rater’s personal estimation of small bowel % remaining, ICCs are presented with their 95% CI. The values of the Cohen’s kappa coefficient and the ICC are presented with their 95% CI.

#### Percentage of the remaining small bowel

For both raters, two variables (the number and the percentage of noninvaded small bowel arteries) were statistically correlated with the percentage of small bowel remaining after surgery (*P* < 0.05) (Table 3).

Correlation coefficients were positive for both raters for both the number and the percentage of noninvolved SMA small bowel branches. Concerning the percentage of noninvolved SMA small bowel branches, the correlation coefficients (*ρ*) were 0.74 for the senior rater and 0.63 for the resident rater. About the rater’s personal estimation of the percentage of the remaining small bowel, the ICCs were 0.64 (95% CI: 0.42–0.78) for the senior rater and 0.50 (95% CI: 0.25–0.70) for the resident rater.

### Inter-rater reliability analysis

All the CT variables studied were subjected to an interobserver reproducibility study.

For Si-NET multiplicity, the presence of a mesenteric mass, right colic resection, duodenal resection and retropancreatic LN involvement, the kappa coefficients are presented in Table 2. For the assessment of the length and percentage of the remaining small bowel, data for the four signs tested are presented in Table 3.

The interobserver reproducibility was very good for the number and the percentage of noninvolved SMA small bowel branches, with an ICC of 0.80 (95% CI: 0.64–0.89) and 0.82 (95% CI: 0.69–0.90).

In contrast, the ICC was low for rater’s personal estimation of the percentage of the remaining small bowel, measured at 0.58 (95% CI: 0.34–0.74).

## Discussion

Surgery of Si-NETs is challenging because of the mesenteric tumor extension that can lead to a postoperative short small bowel syndrome due to vascular invasion (Pasquer *et al.* 2021). Recent anatomopathological studies have distinguished LN metastases from MTDs, which are the cause of arterial vascular involvement of the branches of the SMA (Gonzalez *et al.* 2018). Radiological work has so far mainly focused on the primary tumors (Soyer *et al.* 2013), and the most recent research on the mesenteric extension has targeted LN involvement (Lardière-Deguelte *et al.* 2016, Gupta *et al.* 2022). The present study evaluates preoperative CT performances and reproducibility in the surgical planning of Si-NETs in light of this novel pathological concept of MTDs.

In this study, we have assessed the mesenteric extension of Si-NETs, based on a previous classification (Lardière-Deguelte *et al.* 2016), conceived before the introduction of MTDs in the American Joint Committee on Cancer (AJCC) Cancer Staging Manual, which had already introduced the notion of level of SMA involvement based on the position of mesenteric LN metastases. Interestingly, there was no correlation between the number of removed LNs and the length of small bowel resection. Replacing evaluation of LN location by that of MTD location in our study showed that the level of invasion of the SMA is statistically associated with the length and percentage of the remaining small bowel after surgery with a very good reproducibility (*ĸ* = 0.78) between radiologists. The percentage of SMA small bowel branches noninvolved by the mesenteric mass is statistically associated with the percentage of the remaining small bowel with a high correlation coefficient (between 0.63 and 0.74) and with a very good inter-rater reliability (ICC = 0.82). This is interesting, as the personal estimations by the raters were globally inaccurate and subject to wide variation; objective data seem a better choice to estimate the percentage of the remaining small bowel. The number of SMA small bowel branches noninvolved is also correlated with the length and percentage of the remaining small bowel, but the strength is weaker (correlation coefficient, respectively, between 0.44 and 0.56 and between 0.51 and 0.68), which is related to the variability in small bowel length in our population (320–850 cm) leading to a variability in the number of arterial branches.

We have chosen to focus on the length of the remaining small bowel and not on the resected length, as it seems to be more clinically relevant, the aim of the surgery being to avoid the postoperative short small bowel syndrome.

Our study is the first to test CT in the detection of Si-NET(s)-associated mesenteric mass (MTD > 2 cm), with an excellent sensitivity (97%) and a good reproducibility (*κ* = 0.73). Identification of the mesenteric mass allowed assessing the risk of duodenal resection when it is located in contact with the duodenum: the CT has low PPV but a very high NPV (97–100%) with a very good inter-rater reliability (*κ* = 0.78). Therefore, the persistence of a fat plane between the mesenteric mass and the duodenum almost systematically excludes the need for a duodenal resection. These data must be put into perspective, given the low number of cases of duodenal invasion in our study.

Our study is also the first to correlate multiple CT criteria with the need of right colectomy associated with small bowel resection: in addition to the presence of a tumor near the ileocecal valve, which is a known criterion (Selberherr *et al.* 2017), we tested two other CT signs (the presence of right mesocolon LN metastases and the involvement of the ileocolic artery by the mesenteric mass). The presence of at least one of the three criteria presents a very good PPV (87%) for the evaluation of the need for a right colectomy, with a perfect reproducibility. These results reinforce the need for the detection of the mesenteric mass and the associated vascular invasion (which may concern small bowel branches but also right colic branches) and the need for a systematic analysis of the right mesocolon on CT in the assessment of locoregional extension of Si-NETs.

CT sensitivity for retropancreatic LN involvement is low (24%), despite a high interobserver reproducibility (*ĸ* = 0.88), and CT cannot be utilized to predict retropancreatic LN involvement. This is important as retropancreatic LN involvement is not always associated with mesenteric LN involvement (Pasquer *et al.* 2016).

Even with respect to the STARD guidelines for diagnostic accuracy studies (Bossuyt *et al.* 2015), this study has limitations. We conducted a single-center retrospective study, which only includes patients who underwent surgery of Si-NETs. It was therefore impossible to define criteria for tumor resectability. Moreover, the validation of our CT criteria requires prospective studies from high-volume centers, with more patients.

In conclusion, our study shows that CT is accurate in the identification of the mesenteric mass associated with small intestine neuroendocrine tumor(s), which is the cornerstone of surgical planning, because it is responsible for the superior mesenteric vascular invasion, which is the key limiting factor for surgery.

Adapting existing classification by focusing on mesenteric mass rather than lymphadenopathy shows that the level of arterial vascular invasion along the superior mesenteric axis allows one to anticipate the length and the percentage of the remaining small bowel. CT also allows one to anticipate, with a good reproducibility, the need for a right colectomy.

## Declaration of interest

There is no conflict of interest that could be perceived as prejudicing the impartiality of the work reported.

## Funding

This research did not receive any specific grant from any funding agency in the public, commercial or not-for-profit sector.
